# Overexpression of the Rv0805 phosphodiesterase elicits a cAMP-independent transcriptional response^☆^

**DOI:** 10.1016/j.tube.2013.05.004

**Published:** 2013-09

**Authors:** Nishad Matange, Debbie M. Hunt, Roger S. Buxton, Sandhya S. Visweswariah

**Affiliations:** a Department of Molecular Reproduction, Development and Genetics, Indian Institute of Science, Bangalore 560012, India; b Division of Mycobacterial Research, MRC National Institute for Medical Research, Mill Hill, London NW7 1AA, UK

**Keywords:** Cyclic AMP, Phosphodiesterase, Microarray, Rv0805, *Mycobacterium tuberculosis*

## Abstract

The *Rv0805* gene in *Mycobacterium tuberculosis* encodes a metallophosphoesterase which shows cAMP-hydrolytic activity. Overexpression of Rv0805 has been used as a tool to lower intracellular cAMP levels and thereby elucidate the roles of cAMP in mycobacteria. Here we show that levels of cAMP in *M. tuberculosis* were lowered by only ∼30% following overexpression of Rv0805, and transcript levels of a number of genes, which include those associated with virulence and the methyl citrate cycle, were altered. The genes that showed altered expression were distinct from those differentially regulated in a strain deleted for the cAMP-receptor protein (CRP^Mt^), consistent with the relatively low dependence on cAMP of CRP^Mt^ binding to DNA. Using mutants of Rv0805 we show that the transcriptional signature of Rv0805 overexpression is a combination of catalysis-dependent and independent effects, and that the structurally flexible C-terminus of Rv0805 is crucial for the catalysis-independent effects of the protein. Our study demonstrates the dissociation of Rv0805 and cAMP-regulated gene expression, and reveals alternate functions for this phosphodiesterase from *M. tuberculosis*.

## 1 Introduction

Cyclic adenosine monophosphate (cAMP) is a key modulator of bacterial physiology regulating a variety of processes ranging from carbon metabolism to virulence. Bacteria of the genus *Mycobacterium*, including the pathogen *Mycobacterium tuberculosis*, are known to produce and secrete large amounts of cAMP during planktonic growth.^1,2^ Intracellular cAMP levels in mycobacteria are also modulated by growth conditions such as pH,^1,3^ membrane perturbation^1^ and nutrient content,^1^ providing evidence for the signaling potential of this second messenger. The roles of cAMP in colonization of the host by *M. tuberculosis*^4,5^ suggest that a study of cAMP-signaling mechanisms in mycobacteria is relevant.

The genome of *M. tuberculosis* H37Rv codes for 16 biochemically diverse adenylyl cyclases^6,7^, of which Rv0386,^8^ Rv1264,^9^ Rv1625c,^10^ Rv1900c,^11^ Rv2212,^12^ Rv1647,^13^ Rv1318c, Rv1319c, Rv1320c and Rv3645^14^ have activity *in vitro*. Domain composition and data on activators and inhibitors of these proteins suggest that cAMP synthesis by these enzymes is regulated by a variety of environmental cues such a fatty acids,^12^ polyphosphates^15^ and pH.^16^ Ten putative cAMP-binding proteins, which act as effectors of cAMP-signaling, are also encoded by *M. tuberculosis* H37Rv.^6,7^ Characterized among these are the cAMP-regulated protein acetylase, Rv0998,^17^ and the cAMP-regulated CRP-like transcription factors Rv3676^18^ and Rv1675c.^3^ Of the two CRP-like transcription factors in *M. tuberculosis* direct binding of cAMP has been demonstrated only for Rv3676^19^ (henceforth CRP^Mt^). Elucidation of the CRP^Mt^ regulon in *M. tuberculosis*^18,20^ and other mycobacteria,^21^ coupled with transcriptomic profiling of *M. tuberculosis* deleted for *CRP*^*Mt*^, have identified several target genes regulated by this protein, such as the resuscitation promoting factor *rpfA*, the transcription factor *whiB1* and the *espACD* genes, components of the ESX-1 secretion system.^20,22^ In accordance with its role as a master regulator of gene expression, *M. tuberculosis* deleted for *CRP*^*Mt*^ showed compromised growth *in vitro*.^20^ Recent studies show that this effect of *CRP*^*Mt*^ deletion could in part be attributed to serine auxotrophy.^23^ *CRP*^*Mt*^ deletion also resulted in compromised virulence of *M. tuberculosis*^20^ highlighting the critical importance of this transcription factor in mycobacterial physiology and pathogenesis.

The role of cAMP phosphodiesterases (like *cpdA* from *Escherichia coli*) in modulating intracellular cAMP levels in multiple species of bacteria is well documented.^24–26^ In contrast to the large number of genes that encode nucleotidyl cyclases in mycobacteria, only a single cAMP phophodiesterase, coded by the *Rv0805* gene, was identified in the genome of *M. tuberculosis* H37Rv.^27^ This gene has close orthologs only in genomes of bacteria from the *M. tuberculosis*-complex, and is absent from the genomes of fast growing mycobacteria. *In vitro* biochemical characterization showed that Rv0805 is an active phosphodiesterase capable of hydrolyzing not only 3′5′-cAMP, but also other substrates such as 2′3′-cAMP and bis-p-nitrophenol phosphate.^28^ Structural and biochemical evidence have highlighted the importance of metal binding in catalysis, and identified Asn97, a metal binding residue in the active site of Rv0805, as being critical for cAMP hydrolysis *in vitro*^28,29^ and *in cellulo*.^28^ The structurally flexible C-terminus of Rv0805, while not essential for catalysis, is involved in building the active site of Rv0805, since deletion of the C-terminus resulted in poorer utilization of linear phosphodiester substrates.^28^

Overexpression of full length Rv0805(1–318) in *M. tuberculosis* has been used to lower cAMP levels in cells, in order to elucidate the role of bacterially derived cAMP in attenuating the response of the macrophage.^4^ We have reported earlier that overexpression of Rv0805(1–318) in *Mycobacterium smegmatis*^28^ did indeed lead to a decrease in intracellular cAMP levels, demonstrating the activity of this enzyme against cAMP *in vivo*. However, Rv0805 overexpression in *M. smegmatis* also resulted in increased sensitivity to factors that perturbed the cell wall.^28^ Curiously, this phenotype was independent of the cAMP-hydrolytic activity of Rv0805, since overexpression Rv0805(1-278), that lacks the C-terminus of Rv0805 but is still capable of hydrolyzing cAMP, did not result in this phenotype.^28^

In this study we show that Rv0805 overexpression elicits a transcriptional response that is independent of its ability to hydrolyze cAMP. The gene expression profile in Rv0805 overexpression strains differs from that seen in the Δ*CRP*^*Mt*^ strain, indicating a divergence in the functions of these two proteins, and possible catalysis-independent functions of this phosphodiesterase.

## 2 Material and methods

### 2.1 Strains and plasmids

*M. tuberculosis* H37Rv strains and plasmids used in this study are listed in Table 1. *M. tuberculosis* H37Rv was grown in Dubos Medium (supplemented with 5% Dubos Medium albumin and 0.2% glycerol) in roller bottles at 37 °C. Hygromycin B was included at a final concentration of 50 μg/mL wherever necessary.

### 2.2 Overexpression of Rv0805 in M. tuberculosis

The *Rv0805* gene along with its promoter was amplified from the genome of *M. tuberculosis* H37Rv by PCR using primers Rv0805promfwd (Table 2) and Rv0805rvs.^27^ The PCR product was digested with *Xba*I and *Sac*I to yield a fragment (fragment I) containing the *Rv0805* promoter and part of the protein coding region of the gene and cloned into similarly digested pBKS-II(+) vector to yield pBKS-Rv0805-fragment I. The sequence of the cloned fragment was confirmed. Fragment I was then released using *Xba*I and *Sac*I and ligated, along with a *Sac*I–*Hin*dIII digested fragment derived from pPRO-Rv0805^1-318^,^27^ into *Xba*I and *Hin*dIII cut pMV-10-25 to yield pMV-*Rv0805*prom-*Rv0805*. Mutants Rv0805N97A and Rv0805(1-278)N97A were cloned similarly using the *Sac*I–*Hin*dIII fragments from pBKS-Rv0805N97A and pBKS-Rv0805(1-278)N97A plasmids respectively. All plasmids were electroporated into electrocompetent *M. tuberculosis* H37Rv and transformants were selected on hygromycin containing 7H10 agar. Transformed bacteria were grown in Dubos broth in roller bottles at 37 °C. Rv0805 overexpression was confirmed by Western blotting using lysates from mid-log phase cultures. Equal protein (20–50 μg) from lysates of all strains was subjected to SDS-PAGE and blotted onto polyvinylidene difluoride membranes (Millipore). Rv0805-specific monoclonal antibody culture supernatants^28^ or Rv0805-specific polyclonal antiserum were used at 1:50 or 1:5000 dilution respectively. Bound antibody was detected using chemiluminescence (ECL Plus, GE Healthcare).

### 2.3 Measurement of cAMP levels in M. tuberculosis

For measurement of intracellular cAMP levels, *M. tuberculosis* harboring plasmids overexpressing Rv0805 or the empty vector were grown in Dubos broth. Aliquots were taken during the logarithmic phase of growth and centrifuged to separate cells from the growth medium. Cell pellets were resuspended in 0.1 N HCl and heated for 5 min at 95 °C. Samples were stored at −70 °C until further use. Cyclic AMP in HCl extracts was measured using a cAMP-ELISA kit (Enzo Life Science) according to the manufacturer's instructions or by radioimmunoassay as described earlier.^1^

### 2.4 Transcription analysis using DNA microarrays

*M. tuberculosis* strains harboring Rv0805 overexpression constructs or empty vector were grown in Dubos broth in roller bottles at 37 °C. Bacteria were harvested at mid-log phase (light transmittance at 600 nm ∼ 0.6). Total RNA was extracted using a Fast RNA Pro Blue kit (MP Biomedicals). RNA was treated with RNase-free DNase (Promega) and purified using RNAeasy columns (Qiagen) according to the manufacturer's instructions. Fluorescently labeled cDNA was generated from total RNA (5–10 μg) by direct incorporation of Cy3- or Cy5-dCTP (GE Healthcare) using Superscript II Reverse Transcriptase (Invitrogen Life Technologies) in the presence of random hexamers (3 μg), dNTPs (185 μM dCTP and 463 μM each of dATP, dGTP and dTTP) and Cy3-dCTP or Cy5-dCTP (1.7 nmoles). RNA and random hexamers were initially mixed, made up to a volume of 11 μL and heated to 95 °C for 5 min. The mixture was then chilled on ice for 2 min. Remaining components of the reverse transcriptase reaction were added and the mixture was incubated for 10 min at 25 °C, followed by 42 °C for 90 min. Samples to be compared were mixed and the labeled cDNA was purified using MinElute PCR purification columns (Qiagen). *M. tuberculosis* whole genome microarray slides (prepared at St. George's, University of London) were initially prehybridized in 3.5× SSC, 0.1% SDS and 10 mg/mL BSA for 20 min at 65 °C and then washed with deionized water and isopropanol. Labeled samples were heated for 2 min at 95 °C in 4xSSC and 0.3% SDS and hybridized on microarray slides under Lifter Slips (Thermo Scientific) for 16–20 h at 65 °C in a dark hybridization chamber. Hybridized slides were washed once with 1XSSC, 0.05% SDS at 65 °C and then twice with 0.06xSSC at room temperature. Washed slides were dried by centrifugation and scanned for fluorescence with a GenePix 4000B microarray scanner. Grids were fitted to the raw microarray images, and background normalization and spot quantitation was performed using Bluefuse software (BlueGnome Ltd., Cambridge, United Kingdom) and normalized readings were plotted using GeneSpring 10 software (Silicon Genetics). Data were obtained for six slides, including dye swaps, from three bacterial cultures. Data were initially filtered on expression and the lower 20th percentile was eliminated from the analysis. Genes that showed >2-fold change in absolute expression with a *p*-value <0.05 (Student's *t*-test) were considered to be altered. Raw data from microarrays for the *CRP*^*Mt*^ knockout published previously^20,22^ were re-analyzed similarly for comparison with the Rv0805 overexpression data set.

### 2.5 Microarray data accession numbers

Fully annotated microarray data have been deposited in BμG@Sbase (accession number E-BUGS-146; http://bugs.sgul.ac.uk/E-BUGS-146) and also ArrayExpress (accession number E-BUGS-146). The array design is available in BμG@Sbase (Accession No. A-BUGS-23; http://bugs.sgul.ac.uk/A-BUGS-23) and also ArrayExpress (Accession No. A-BUGS-23).

### 2.6 Real-time quantitative PCR

Real-time quantitative PCR was carried out using SYBR Green Master Mix (Applied Biosystems) on an ABI Prism 7700 Sequence detection system. *rrs*, coding for 16S rRNA, was used for normalization. Primers used in this study are listed in Table 2. Gene specific primers for Real-time PCR analysis were designed using the Primer Express software (PE Applied Biosciences).

## 3 Results

### 3.1 Overexpression of Rv0805 in M. tuberculosis under its endogenous promoter

**Figure 1:**
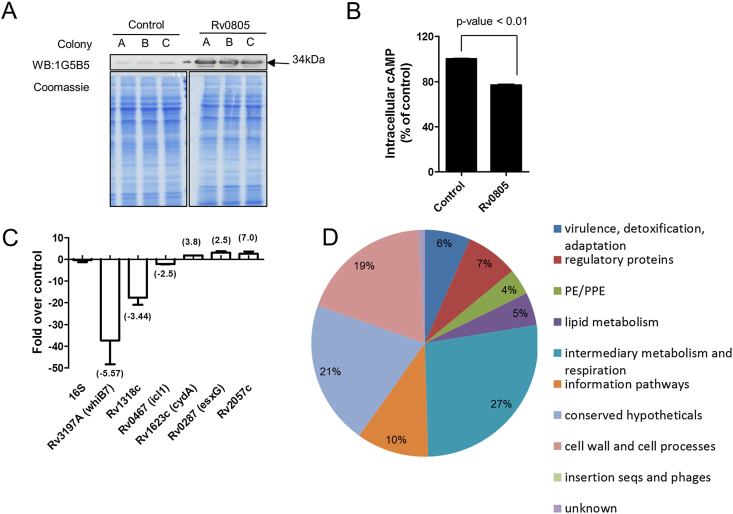
Overexpression of Rv0805 elicits changes in gene expression. A. Confirmation of Rv0805 overexpression in *M. tuberculosis* by immunoblotting using Rv0805-specific antibodies. Equal amounts of protein from cell lysates of strains containing empty vector (Control) or Rv0805 overexpression constructs (Rv0805) were used for western blotting. Robust overexpression of wild type Rv0805 was observed in three independent transformants (colonies A, B, C). B. Measurement of intracellular cAMP levels in *M. tuberculosis* overexpressing Rv0805 (Rv0805) compared to the empty vector (control). Bacterial cAMP was measured in the exponential phase of growth (Light transmittance at 600 nm ∼ 0.6). Expression of wild type Rv0805 led to decreased intracellular cAMP levels. Mean ± SEM of measurements done in triplicates from three independent biological replicates is plotted. C. Validation of microarray results by qRT-PCR was performed for three up-regulated (*Rv1623c*, *Rv0287* and *Rv2057c*) and three down-regulated genes (*Rv3197A*, *Rv1318c* and *Rv0467*). *rrs*, coding for 16S rRNA, was used as the normalizing gene. Bars represent mean ± SEM of expression level of genes in *M. tuberculosis*-Rv0805 relative to *M. tuberculosis*-VC as measured from triplicate cultures. Numbers in parentheses represent fold regulation from microarray results. D. Analysis of cellular pathways affected by Rv0805 overexpression. Genes with altered expression levels (>±2 fold) on expression of Rv0805 were assigned functional categories according to Tuberculist (www.tuberculist.epfl.ch) annotation. Numbers represent percentage of total altered genes.

We initially attempted to overexpress Rv0805 in *M. tuberculosis* under the *Mycobacterium bovis hsp60* promoter, but found substantial heterogeneity in the level of overexpression between transformants. Overexpression was also temporally unstable (not shown). As an alternative to the *hsp60* promoter, we therefore chose to overexpress Rv0805 under its own promoter. The region upstream of *Rv0805* (∼500 bps) has been used previously to overexpress the adenylyl cyclase Rv1264 in *M. bovis* BCG and *M. tuberculosis*.^3^ Since the promoter region used in this earlier study^3^ contained a large part of the *Rv0804* open reading frame, we attempted to use a shorter DNA sequence limited to the intergenic region between *Rv0805* and *Rv0804* (∼150 bps) to overexpress Rv0805 from a multi-copy plasmid. Immunoblotting confirmed consistent overexpression of Rv0805 in *M. tuberculosis* from three independent transformants (Figure 1A). The intracellular cAMP level was reduced on expression of Rv0805 compared to control bacteria (Figure 1B), and the extent of reduction in intracellular cAMP was ∼30%, in agreement with earlier observations.^4,28^

### 3.2 Transcriptional changes on overexpression of Rv0805

Rv0805 overexpression in *M. tuberculosis* has been used previously to reduce intracellular cAMP levels.^4^ It was also demonstrated that infection with Rv0805 overexpressing *M. tuberculosis* elicited lower phosphorylation of CREB and impaired TNF-α production by macrophages, in comparison with wild type bacteria,^4^ suggesting a reduced immunomodulatory capacity of this strain. In order to understand the nature of pathways perturbed by Rv0805 overexpression in *M. tuberculosis*, we analyzed the transcriptome of *M. tuberculosis* overexpressing Rv0805 (*M. tuberculosis*-Rv0805). *M. tuberculosis* harboring the empty vector (*M. tuberculosis*-VC) was used as the reference strain. Rv0805 transcript levels were 36-fold higher in *M. tuberculosis*-Rv0805 than *M. tuberculosis*-VC, thus validating its overexpression (see also Figure 1A, 3A). Additionally, transcript levels of 107 genes were altered significantly (Supplementary Table 1) of which 88 (84%) were down-regulated in *M. tuberculosis*-Rv0805. Using quantitative RT-PCR, the transcript levels of six representative genes were analyzed and found to be in agreement with results from our microarray analysis (Figure 1C).

Differentially regulated genes (which we call Gene List 1) were classified on the basis of functional categories as annotated by Tuberculist^30^ (Figure 1D). While no overwhelming bias was observed, genes involved in ‘intermediary metabolism and respiration’ appeared to be highly represented (21%) in this gene cohort. In addition ‘cell wall and cell processes’ related genes (19%) also showed altered regulation upon overexpression of Rv0805.

### 3.3 Rv0805 induced transcriptional dysregulation is independent of the cAMP-CRP^*Mt*^ pathway

**Figure 2:**
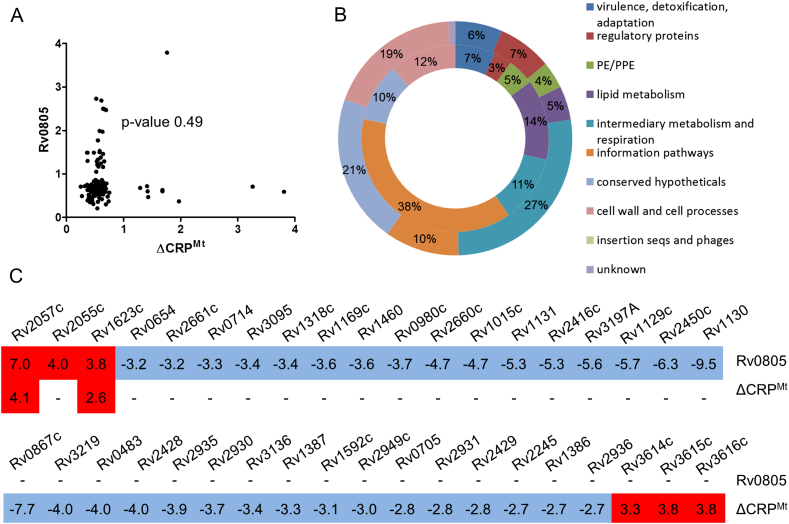
Comparison of changes in the transcriptome following Rv0805 overexpression and in the Δ*crp* mutant A. Comparison of Rv0805 overexpression gene set and the CRP^Mt^ regulon.^20,22^ Fold regulation of 160 genes from the two data sets were compared on a scatter plot. Pearson's correlation analysis was performed using Graphpad Prism and was found not to be statistically significant (*p*-value 0.49). B. Comparison of cellular pathways affected by Rv0805 overexpression (outer donut) and CRP^Mt^ deletion (inner donut). Genes with altered expression levels (>±2 fold) were assigned functional categories according to Tuberculist (www.tuberculist.epfl.ch) annotation. Numbers represent percentage of total genes altered. C. Comparison of genes altered by Rv0805 overexpression and *CRP*^*Mt*^ deletion. Genes dysregulated upon Rv0805 overexpression (upper panel) do not show significant alteration in expression levels in the *CRP*^*Mt*^ knockout and vice-versa (lower panel). Red boxes indicate up-regulation and blue boxes indicate down-regulation compared to appropriate reference strains. Numbers indicate fold change in expression level as obtained from microarray analyses. ‘-’ indicates no change in expression levels (<2.0 fold).

In order to correlate altered gene expression in *M. tuberculosis*-Rv0805 with reduced intracellular cAMP levels, we compared our data set (Gene List 1) with genes dysregulated in a *CRP*^*Mt*^ knockout strain.^20,22^ Since CRP^Mt^ is a cAMP-regulated transcription factor, it was expected that similar genes would be dysregulated upon deletion of *CRP*^*Mt*^ and cAMP-depletion by overexpression of Rv0805. However, there was a poor correlation (*p*-value 0.49) between the CRP^Mt^ regulon and results from the Rv0805 overexpression microarray (Figure 2A). In agreement with this observation none of the genes showing altered expression in *M. tuberculosis*-Rv0805 had predicted upstream CRP^Mt^ binding sites.^18,21,22^ In addition, the cellular pathways affected in the *CRP*^*Mt*^ deletion strain differed from those affected on Rv0805 overexpression (Figure 2B). This suggested that changes in transcription upon Rv0805 overexpression were independent of cAMP/CRP^Mt^. Closer analysis showed that none of the genes differentially regulated in the *CRP*^*Mt*^ knockout strain showed significant alteration in expression levels upon Rv0805 overexpression (Figure 2C), and vice-versa. Two genes *Rv1623c* and *Rv2057c* did show similar trends in the *CRP*^*Mt*^ knockout and Rv0805 overexpression strains (Figure 2C). However, since neither of these genes have upstream predicted CRP^Mt^ binding sites,^18,21^ this may reflect secondary or indirect effects. Thus, the primary effects of Rv0805 overexpression on the cellular transcriptome were not due to perturbation of the cAMP/CRP^Mt^ pathway.

### 3.4 Catalysis-dependent and independent effects of Rv0805 overexpression

**Figure 3:**
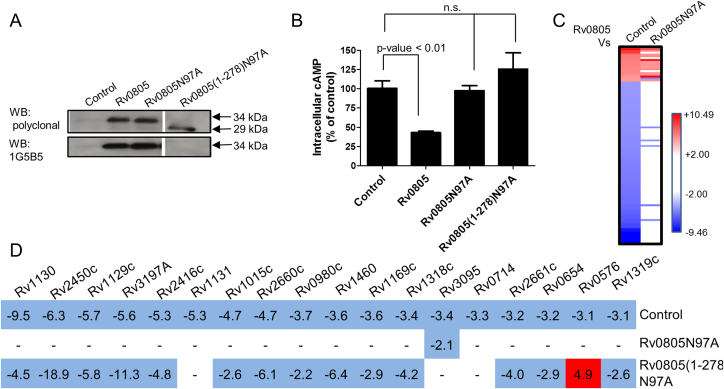
A. Role of the C-terminus of Rv0805 in gene expression changes. Overexpression of Rv0805N97A and Rv0805(1-278)N97A. Immunoblot for lysates of *M. tuberculosis* harboring empty vector (control) or plasmid for overexpression of Rv0805, Rv0805N97A or Rv0805(1-278)N97A. Polyclonal antibodies against Rv0805 or a C-terminus specific monoclonal antibody (1G5B5) were used to distinguish between the full length and C-terminally truncated proteins based on differential mobility and loss of immunoreactivity respectively. B. Intracellular cAMP levels in *M. tuberculosis* overexpressing Rv0805, Rv0805N97A or Rv0805(1-278)N97A. Mean ± SEM of at least two biological replicates are plotted. (n.s. indicates that the two groups compared are statistically not significant (*p*-value > 0.05)) C. Heat map showing expression levels of genes in *M. tuberculosis*-Rv0805 compared with a strain harboring empty vector (control) or overexpressing Rv0805N97A. Two distinct gene sets (A and B) are clearly discernible. D. Genes showing altered regulation in the Rv0805 overexpression strain compared to *M. tuberculosis* harboring empty vector (control) or overexpressing Rv0805N97A or Rv0805(1-278)N97A. Presence of the C-terminus of Rv0805 is essential for manifestation of catalysis-independent gene expression changes. Red boxes indicate up-regulation and blue boxes indicate down-regulation in *M. tuberculosis*-Rv0805 compared to the indicated strains. Numbers indicate fold change in expression level as obtained from microarray analyses. ‘-’ indicates no change in expression levels (<2.0 fold).

We have shown earlier that Rv0805 overexpression in *M. smegmatis* resulted in cell wall perturbation in a cAMP hydrolysis-independent manner.^28^ To address if the observed transcriptomic changes in the present study were dependent on cAMP hydrolysis, we directly compared the transcriptomes of *M. tuberculosis*-Rv0805 and *M. tuberculosis* overexpressing Rv0805N97A (*M. tuberculosis*-N97A), a catalytically inactive mutant of Rv0805^28^ that does not reduce intracellular cAMP levels when overexpressed in *M. smegmatis*.^28^ Expression levels of wild type and Rv0805N97A mutant proteins in *M. tuberculosis* were similar as assessed by immunoblotting (Figure 3A), but no reduction in intracellular cAMP levels was seen in *M. tuberculosis* overexpressing Rv0805N97A (Figure 3B).

We compared Gene List 1 with the genes that were differentially regulated in the current microarray (Gene List 2, Supplementary Table 2). Two cohorts of genes were identifiable based on this analysis (Figure 3B). The first cohort of genes (gene set A, Supplementary Table 4) was dysregulated in both gene lists, implying a strict dependence on the catalytic activity of overexpressed Rv0805. The second cohort of genes (gene set B, Supplementary Table 5) showed altered expression in Gene list 1 but not in Gene list 2, implying catalysis-independent effects of Rv0805 overexpression (Figure 3C). A third set of genes were present only in Gene List 2 but not Gene List 1 (Supplementary Table 6), suggesting that their dysregulation was due to overexpression of catalytically inactive Rv0805N97A alone. Most of these genes belonged to the DosR regulon, including *dosR* itself. Close inspection of the data revealed that one of the three *M. tuberculosis*-Rv0805 biological replicates also showed altered expression of this set of genes. Since the DosR regulon is induced by multiple stresses such as hypoxia,^31^ exposure to ethanol and even centrifugation^32^ its down-regulation in *M. tuberculosis* overexpressing Rv0805 may indicate a cellular stress-response to protein overproduction. We therefore excluded these genes from further analysis.

We analyzed Gene sets A and B in order to reveal specific pathways affected in each case.

### 3.5 Analysis of Gene set A

#### 3.5.1 ESX systems and metal regulated genes

The ESX-3 system, one of 5 type VII secretion systems in *M. tuberculosis* was up-regulated exclusively on expression of wild type Rv0805 (Rv0286 +2.4, Rv0287 +2.5, Rv0288 +2.7, Rv0289 +2.7, Rv0290 +2.5). This system is thought to play a role in metal acquisition^33^ and is induced in low iron conditions by the IdeR transcriptional regulator.^34^ Studies have also shown the regulation of this operon by Zur, a zinc dependent transcription factor.^35^ Two other operons regulated by Zur were also up-regulated in this category of genes. These are *esxR* (Rv3019c +2.5) and *esxS* (Rv3020c +2.4), secreted antigens similar to ESAT-6 and Cfp-10, and *Rv2057c*, the gene encoding the ribosomal protein Rv2057c (Rv2057c +7.0).^35^

### 3.6 Analysis of Gene set B

#### 3.6.1 Cell wall associated genes

Several genes that are associated with cell wall processes were identified in this gene set. The resuscitation promoting factor E (*rpfE*; Rv2450c -6.3) showed maximal down-regulation. Rpfs are thought to be associated with the process of reactivation of the tubercle bacillus.^36^ While dispensable for growth of mycobacteria *in vitro*, they are required for complete virulence in the mouse model.^36^ In particular, *rpfE* was induced by low pH and hypoxia, both thought to be stresses experienced by *M. tuberculosis* in the host.^37^

In addition to *rpfE*, *iniA* (Rv0342 -2.6) and *iniB* (Rv0341 -2.8) were also down-regulated on Rv0805 overexpression. Both of these two genes along with *iniC* were identified as genes induced by the cell wall targeting drug isoniazid.^38^ IniA and B are thought to be components of a drug efflux system that allows increased tolerance toward isoniazid and ethambutol.^39^ In addition mutations were identified in these genes in isoniazid-resistant isolates of *M. tuberculosis*.^40^

#### 3.6.2 Adenylyl cyclases Rv1318c and Rv1319c

Among the genes down-regulated on Rv0805 overexpression were the adenylyl cyclases *Rv1318c* (-3.4) and *Rv1319c* (-3.1), which along with *Rv1320c* form part of a putative operon. These cyclases are membrane bound and contain a signaling HAMP domain that regulates the activity of the cyclase domain.^6,41^ Transcript levels of other adenylyl cyclases were unaltered suggesting that the down-regulation of *Rv1318c* and *Rv1319c* was not a general response due to perturbation of the cAMP homeostasis in the cell.

#### 3.6.3 Genes of the methyl citrate cycle

Amongst the most highly down-regulated genes in this gene set were *Rv1129c* (-5.7), *Rv1130* (*prpC*; -9.5) and *Rv1131* (*prpD*; -5.3). Rv1129c is a transcriptional activator leading to enhanced transcription of *prpC* and *prpD*, both components of the methyl citrate cycle.^42^ In addition to these *Rv0467* (*icl1*; -2.5), another enzyme from the methyl citrate shunt,^43^ was also down-regulated. Thus overexpression of Rv0805 led to a decrease in transcript levels of the entire methyl citrate cycle.

#### 3.6.4 WhiB7 and Eis

Both *whiB7* (*Rv3197A* -5.6) and its downstream target, *eis* (*Rv2416c* -5.3), were down-regulated in *M. tuberculosis*-Rv0805. WhiB7 is one of 7 WhiB-like proteins found in *M. tuberculosis*, and has been associated with tolerance toward free fatty acids and some antimicrobials.^44^ Recent studies have also shown that levels of WhiB7 in the cell are regulated by the redox potential of the cell.^45^ Eis is an acetyl-transferase that when overexpressed in *M. smegmatis* leads to increased virulence.^46^ Due to its ability to acetylate a wide range of aminoglycosides, Eis has been linked to resistance of XDR-TB to kanamycin.^47^ It has also been associated with increased virulence of the Beijing strain of *M. tuberculosis*.^48^ The dysregulation of the *whiB7-eis* axis on Rv0805 overexpression is of particular interest given the virulence-related phenotypes of both Rv0805 deletion and overexpression. It is also pertinent that the transcript level of these genes were altered independently of the catalytic activity of Rv0805, reiterating that Rv0805 overexpression may alter virulence-related pathways unrelated to its cAMP-hydrolytic ability.

### 3.7 Effects of the C-terminus on catalysis-dependent and independent effects

Our earlier studies using *M. smegmatis* showed cAMP hydrolysis-independent perturbation of the cell wall upon overexpression of Rv0805.^28^ The flexible C-terminus of the protein mediated this phenotype as overexpression of Rv0805(1-278), which lacks the last 40 amino acids, did not affect cell wall properties of *M. smegmatis*.^28^ Based on these studies we asked if the presence of the C-terminus of Rv0805 contributed to the gene expression changes observed in *M. tuberculosis* as well, by attempting to overexpress Rv0805(1-278). We were unable to obtain strains that overexpressed wild type Rv0805(1-278), but were successful in obtaining a strain of *M. tuberculosis* overexpressing Rv0805(1-278)N97A, confirmed by western blotting by a change in mobility of the immunoreactive protein (Figure 3A). The absence of the C-terminus in the overexpressed protein was further verified through a loss of immunoreactivity to a monoclonal antibody specific for the C-terminus of Rv0805 (Figure 3A). Rv0805(1-278)N97A overexpression did not reduce intracellular cAMP levels in *M. tuberculosis*, confirming that it was unable to hydrolyze cAMP *in vitro* (Figure 3B).

We then compared the changes in gene expression between *M. tuberculosis*-Rv0805 overexpressing strains and *M. tuberculosis* expressing Rv0805(1-278)N97A. Genes that were differentially expressed between these two strains (Gene List 3) were inspected for the presence of genes found in gene set B. Gene set B genes showed a similar trend in Gene list 3 as well as Gene list 1, confirming that catalysis-independent transcriptional effects of Rv0805 overexpression required the presence of the C-terminus of Rv0805 (Figure 3D). These results are commensurate with earlier observations^28^ emphasizing the importance of the C-terminus of Rv0805 in orchestrating the *in vivo* activities of the protein.

## 4 Discussion

In this study, we have used DNA microarrays to elucidate gene expression changes associated with overexpression of Rv0805, in *M. tuberculosis* H37Rv. Our data show that Rv0805 overexpression does not alter the levels of cAMP-CRP^Mt^ regulated genes, even though cAMP levels were reduced in cells. Moreover, the genes dysregulated by Rv0805 overexpression do not appear to be targets of CRP^Mt^. Therefore, overexpression of Rv0805 in *M. tuberculosis* does not directly target the cAMP pathway mediated by CRP^Mt^, but instead can regulate the expression of genes, independent of its catalytic activity.

Overexpression of Rv0805 was achieved under its endogenous promoter from a multi-copy plasmid. In addition to robust overexpression, this strategy ensured that the overexpressed protein was similar to the endogenous protein in terms of its translational start site. To our knowledge such an approach to protein overexpression in mycobacteria has not been employed previously. The *hsp60* promoter is widely used for protein overexpression in mycobacteria, but has been shown to be unstable on occasion.^49^ Indeed, our own attempts at overexpressing Rv0805 under the *hsp60* promoter in *M. bovis* BCG met with little success (data not shown). The Rv0805 promoter may therefore provide an alternative to the *hsp60* and *sigA* promoters for protein overexpression in mycobacteria.

Even though Rv0805 overexpression did lead to a reduction in intracellular cAMP levels in *M. tuberculosis*, we did not find alteration in expression levels of CRP^Mt^ regulated genes. This observation reiterates differences seen between the *E. coli* CRP and CRP^Mt^ for cAMP-regulated DNA binding.^19^ The lower affinity for cAMP and lower dependence on cAMP for DNA binding by CRP^Mt^ have been investigated both biochemically^19^ and structurally.^50^ Indeed, higher levels of cAMP in mycobacteria than enteric bacteria such as *E. coli*^1,2^ may have resulted in desensitization of CRP^Mt^ to small changes in intracellular cAMP levels. Given the moderate reduction in cAMP levels upon overexpression of Rv0805, it is likely that additional mechanisms to regulate intracellular cAMP, other than the catalytic activity of Rv0805, may be operative in mycobacteria. Since mycobacteria secrete large amounts of cAMP, efflux of cAMP may be an important mechanism for modulating intracellular cAMP levels in these bacteria. Further, the absence of direct orthologs of Rv0805 in the genomes of fast growing mycobacteria suggest that Rv0805 may not be primarily responsible for cAMP hydrolysis in these bacteria.

*M. tuberculosis* codes for 9 other putative cAMP-responsive proteins^6^ that may directly or indirectly mediate the transcriptional effects of Rv0805 overexpression observed in this study. Two of these, Rv1675c (Cmr) and Rv0998, have been characterized. Cmr is a transcription factor that is thought to be regulated by cAMP; however, direct binding of cAMP to this protein has not been demonstrated to date. Cmr is unlikely to mediate the transcriptional dysregulation observed upon Rv0805 overexpression, as known targets of Cmr (such as Rv1265, groEL2 and mdh^3^) were unaffected in our experiments. Rv0998, also called KAT_mt_, is a cAMP-dependent protein lysine acetyltransferase.^17,51^ KAT_mt_ was recently shown to acetylate several fatty acyl-CoA ligases (FadDs) including acetyl CoA-synthetase.^51^ The ortholog of KAT_mt_ in *M. bovis* BCG (KAT_bcg_) appears to play a role in reducing propionate toxicity, since a strain deleted for *KAT*_*bcg*_ was compromised for growth on propionate.^51^ Given the dysregulation of several genes involved in propionate metabolism in *M. tuberculosis*-Rv0805, it is possible that the effects of Rv0805 overexpression in *M. tuberculosis* were, at least in part, due to perturbation of KAT_mt_ signaling.

The catalysis-dependent effects of Rv0805 overexpression altered transcription of genes that respond to the levels of cellular metals, alluding to a dysregulation of metal ions in the cell. Rv0805 binds Fe^2+^ and Mn^2+^ at its active site.^27^ This may allow it to act as a sink or sensor for these metals in the cell, at least upon overexpression, resulting in altered regulation of Zur/IdeR metal dependent regulons. In agreement with this, Rv0805N97A overexpression did not result in dysregulation of Zur/IdeR regulon genes. The poorer binding of the mutant protein to metal ions was revealed by crystallographic studies showing that Asn97 is involved in Mn^2+^ co-ordination, and its mutation to Ala resulted in a lower occupancy of metal in the active site.^29^ The implications for possible roles of Rv0805 in regulating metal homeostasis remain to be tested.

Among the genes dysregulated by Rv0805 overexpression in a catalysis-independent manner, several pathways that are involved in virulence were identifiable. The genes *Rv1129c*, *prpC* and *prpD* and *icl1* are part of the methyl citrate pathway and are essential for the full virulence of *M. tuberculosis*.^42^ The methyl citrate pathway plays a role in utilization of propionate derived from cholesterol^52^ and odd chained fatty acids.^53^ It has been speculated that *M. tuberculosis* uses cholesterol as a carbon source in the host.^54^ Indeed, knockouts of *prpC*, *prpD* and *icl1* show compromised growth in bone derived macrophages and poor growth on propionate *in vitro*.^42^ Interestingly, transposon insertion mutants of methyl citrate cycle genes as well as Rv0805 were compromised in a screen for cholesterol utilization.^55^ In addition, a transposon insertion in the intergenic region between *Rv0805* and *Rv0804* (i.e. in the *Rv0805* promoter) in an *icl1* knockout genetic background resulted in under-representation in a TraSH screen for propionate toxicity.^56^ These observations clearly indicate a link between the methyl citrate cycle genes and *Rv0805* that has not yet been explored. Altered regulation of the transcriptional regulator *whiB7* and its target *eis* is also of particular interest. Whib7 is thought to play a role in resistance to fatty acids and several antibiotics. An *eis*-deletion mutant also shows compromised growth on cholesterol^55^ further supporting the hypothesis that Rv0805 may have direct or indirect roles in cholesterol metabolism.

The presence of the C-terminus of Rv0805 was essential for the catalysis-independent effects of Rv0805 overexpression on the transcriptome. This observation is in concordance with earlier studies on the phenotypic effects of Rv0805 overexpression in *M. smegmatis*, and can be attributed to the aberrant localization of C-terminus deficient Rv0805 to the cytosol, in contrast to the membrane localization of the full length protein.^28^ Alternatively, the C-terminus of Rv0805 may provide an interaction domain allowing the recruitment of Rv0805 to appropriate complexes.

High-throughput approaches such as gene expression profiling using microarrays are a useful platform for investigating alterations at the level of the whole genome. However, such methodologies can also provide erroneous data that could affect interpretations of biological phenomena. For example, we identified a set of genes that appeared to be dysregulated upon overexpression of Rv0805N97A, but not Rv0805 in *M. tuberculosis* (Gene List B). Several of the maximally dysregulated genes of this group belonged to the DosR regulon. While this result may indeed be a genuine effect of Rv0805N97A overproduction, it is known that the DosR regulon is induced by a variety of stresses, and indeed, by methods used for RNA extraction.^32^ Perhaps protein overproduction, presumably a stressful condition for the cell, may also result in induction of this regulon. However, we believe that using a combination of high-throughput methodologies with appropriate biochemically validated controls, such as those used in this study, can provide robust results in understanding perturbations of complex biological systems.

In summary, disparate gene regulation profiles of the Rv0805 overexpressing strain and the *CRP*^*Mt*^ knockout mutant, together with the dysregulation of a large cohort of genes independent of catalytic activity, suggest that the primary role of Rv0805 in the cell may not be to regulate intracellular cAMP levels. In an earlier study, Rv0805 overexpression in *M. tuberculosis* was used to demonstrate the importance of cAMP in the virulence of *M. tuberculosis*,^4^ and the reduced virulence of the overexpressing strain was attributed to the activity of Rv0805 as a cAMP phosphodiesterase. In the light of our present study, we find that this may not be entirely true, since overexpression of Rv0805 down regulates a number of genes that have been shown to be important for pathogenesis. The roles of Rv0805 in the cell wall and membrane, in addition to the contribution of the C-terminus of Rv0805 to this protein's function *in vivo*, need to be examined further to completely understand the molecular activities and physiological roles of this phosphodiesterase.

## Figures and Tables

**Table 1:** List of strains and plasmids used in this study.

	Description	Reference or source
***M. tuberculosis* strains**		
*M. tuberculosis* H37Rv	Wild type *M. tuberculosis*	
*M. tuberculosis*-VC	*M. tuberculosis* harboring pMV-empty vector	This study
*M. tuberculosis*-Rv0805	*M. tuberculosis* harboring pMV-Rv0805prom-Rv0805	This study
*M. tuberculosis*-N97A	*M. tuberculosis* harboring pMV-Rv0805prom-Rv0805N97A	This study
*M. tuberculosis*-N97A(1–278)	*M. tuberculosis* harboring pMV-Rv0805prom-Rv0805(1-278)N97A	This study
**Mycobacterial shuttle plasmids**		
pMV-empty vector	pMV10-25 derivative used as control	
pMV-Rv0805prom-Rv0805	pMV10-25 derivative used for overexpression of Rv0805 under its own promoter	This study
pMV-Rv0805prom-Rv0805N97A	pMV10-25 derivative used for overexpression of Rv0805N97A under the Rv0805 promoter	This study
pMV-Rv0805prom-Rv0805(1-278)N97A	pMV10-25 derivative used for overexpression of Rv0805(1-278)N97A under the Rv0805 promoter	This study

**Table 2:** List of primers used in this study.

Primer name	Sequence
Rv0805promfwd	5′ ATCTCTAGACGACTGTGATCGCGGGGTCG 3′
rrs-qRTF	5′ AAGAAGCACCGGCCAACTAC 3′
rrs-qRTR	5′ TCGCTCCTCAGCGTCAGTTA 3′
whiB7RTfwd	5′ GGTGATCCCGATCTGTGGTT 3′
whiB7RTrev	5′ TCACACACAGTGTCTTGGCTACCT 3′
icl1RTfwd	5′ CAGCACATCCGCACTTTGAC 3′
icl1RTrev	5′ ATCACCACCGTGGGAACATC 3′
Rv1130RTfwd	5′ CTGATCCGCGGTCTGGTAA 3′
Rv1130RTrev	5′ CAGATTCCGCGGGTTAGGT 3′
Rv2057cRTfwd	5′ CGCAAGAACCGCCGTAAT 3′
Rv2057cRTrev	5′ CGCAGGATCGGGTCGTATT 3′
cydARTfwd	5′ GCAGCAGCCGATGAAGATG 3′
cydARTrev	5′ CGTCAGGACAGAGAAGTTTGGA 3′
esxGRTfwd	5′ GATGCTCATATCCCACAGTTGGT 3′
esxGRTrev	5′ CGACTCCCCCTGGTGAAAC 3′
Rv1318cRTfwd	5′ ACACTCTACGGCCTGATCAACA 3′
Rv1318cRTrev	5′ CGAACTCGGTATGCAGATAGGTT 3′
rpfERTfwd	5′ TGGTCGATCAACACCGGTAA 3′
rpfERTrev	5′ CTCAGCCACCCGGATCTG 3′
