# Supplementary material for: Overexpression of the Rv0805 phosphodiesterase elicits a cAMP-independent transcriptional response
Source: Tuberculosis (Edinb). 2013 Sep;93(5):492–500. doi: 10.1016/j.tube.2013.05.004 (PMC3776917; doi:10.1016/j.tube.2013.05.004)
Supplement: Supplementary file 1 [file mmc1.docx]

Supplementary Table 1

Gene list 1. Genes showing at least two-fold up- or down-regulation on overexpression of Rv0805, compared to empty vector (VC) (p-value < 0.05). Negative numbers represent down-regulation in the Rv0805 overexpressing strain compared to vector control.

| Gene | Fold Change | p-value | Gene function / annotation |
| --- | --- | --- | --- |
| Rv0047c | -2.7 | 0.011 | conserved hypothetical protein |
| Rv0053 | -2.9 | 0.047 | probable 30s ribosomal protein S6 rpsF |
| Rv0079 | 2.6 | 0.051 | hypothetical protein |
| Rv0197 | 2.0 | 0.052 | possible oxidoreductase |
| Rv0251c | -2.1 | 0.009 | heat shock protein hsp (heat-stress-induced ribosome-binding protein a) |
| Rv0286 | 2.4 | 0.050 | PPE family protein |
| Rv0287 | 2.5 | 0.024 | esat-6 like protein esxG (conserved hypothetical protein tb9.8) |
| Rv0288 | 2.7 | 0.011 | low molecular weight protein antigen 7 esxH (10 kda antigen) (cfp-7) (protein tb10.4) |
| Rv0289 | 2.7 | 0.020 | esx-1 secretion-associated protein espG3 |
| Rv0290 | 2.5 | 0.031 | esx conserved component eccd3. probable transmembrane protein. |
| Rv0302 | -3.0 | 0.050 | probable transcriptional regulatory protein (probably tetR/acrR-family) |
| Rv0316 | -2.2 | 0.015 | possible muconolactone isomerase |
| Rv0337c | -2.2 | 0.006 | probable aspartate aminotransferase aspC (transaminase a) (aspAT) |
| Rv0341 | -2.8 | 0.011 | isoniazid inductible gene protein iniB |
| Rv0342 | -2.6 | 0.016 | isoniazid inductible gene protein iniA |
| Rv0465c | -2.3 | 0.006 | probable transcriptional regulatory protein |
| Rv0467 | -2.5 | 0.024 | isocitrate lyase icl1 (isocitrase) (isocitratase) |
| Rv0477 | -2.0 | 0.034 | possible conserved secreted protein |
| Rv0484c | -2.3 | 0.011 | probable short-chain type oxidoreductase |
| Rv0485 | -2.2 | 0.006 | possible transcriptional regulatory protein |
| Rv0576 | -3.1 | 0.023 | probable transcriptional regulatory protein (possibly arsR-family) |
| Rv0651 | -2.6 | 0.016 | probable 50s ribosomal protein L10 rplJ |
| Rv0654 | -3.2 | 0.011 | probable dioxygenase |
| Rv0656c | -2.1 | 0.008 | possible toxin vapC6 |
| Rv0701 | -2.3 | 0.018 | probable 50s ribosomal protein L3 rplC |
| Rv0714 | -3.3 | 0.003 | probable 50s ribosomal protein L14 rplN |
| Rv0715 | -2.3 | 0.017 | probable 50s ribosomal protein L24 rplX |
| Rv0749 | -2.2 | 0.007 | conserved hypothetical protein with pin domain |
| Rv0805 | 36.5 | 0.005 |  |
| Rv0809 | -2.1 | 0.008 | probable phosphoribosylformylglycinamidine cyclo-ligase purM (airS) (phosphoribosyl-aminoimidazole synthetase) (air synthase) |
| Rv0812 | -2.2 | 0.016 | probable amino acid aminotransferase |
| Rv0839 | -3.0 | 0.030 | conserved hypothetical protein |
| Rv0967 | 2.4 | 0.050 | copper-sensitive operon repressor csoR |
| Rv0969 | 2.4 | 0.050 | probable metal cation transporter p-type atpase ctpV |
| Rv0980c | -3.7 | 0.002 | PE-PGRS family protein |
| Rv1014c | -2.6 | 0.050 | probable peptidyl-tRNA hydrolase pth |
| Rv1015c | -4.7 | 0.002 | probable 50s ribosomal protein L25 rplY |
| Rv1046c | -2.1 | 0.004 | hypothetical protein |
| Rv1057 | -2.6 | 0.050 | conserved hypothetical protein |
| Rv1079 | -2.7 | 0.002 | probable cystathionine gamma-synthase met (cgs) (o-succinylhomoserine [thiol]-lyase) |
| Rv1080c | -2.5 | 0.002 | probable transcription elongation factor greA (transcript cleavage factor grea) |
| Rv1103c | -2.1 | 0.024 | possible antitoxin mazE3 |
| Rv1129c | -5.7 | 0.007 | probable transcriptional regulator protein |
| Rv1130 | -9.5 | 0.007 | possible methylcitrate dehydratase prpD |
| Rv1131 | -5.3 | 0.019 | probable methylcitrate synthase prpC |
| Rv1144 | -2.6 | 0.038 | probable short-chain type dehydrogenase/reductase |
| Rv1169c | -3.6 | 0.050 | PE family protein. possible lipase. |
| Rv1305 | 2.0 | 0.050 | probable atp synthase c chain atpE (lipid-binding protein) (dicyclohexylcarbodiimide-binding protein) |
| Rv1317c | -2.8 | 0.002 | probable ada regulatory protein alkA (regulatory protein of adaptative response) (methylated-dna--protein-cysteine methyltransferase) (o-6-methylguanine-dna alkyltransferase) (o-6-methylguanine-dna methyltransferase) (3-methyladenine dna glycosylase ii) |
| Rv1318c | -3.4 | 0.001 | possible adenylate cyclase (atp pyrophosphate-lyase) (adenylyl cyclase) |
| Rv1319c | -3.1 | 0.003 | possible adenylate cyclase (atp pyrophosphate-lyase) (adenylyl cyclase) |
| Rv1374c | -2.0 | 0.035 | hypothetical protein |
| Rv1405c | -2.0 | 0.050 | putative methyltransferase |
| Rv1460 | -3.6 | 0.050 | probable transcriptional regulatory protein |
| Rv1485 | -2.1 | 0.001 | ferrochelatase hemZ (protoheme ferro-lyase) (heme synthetase) |
| Rv1533 | -2.3 | 0.002 | conserved hypothetical protein |
| Rv1535 | -2.1 | 0.050 | hypothetical protein |
| Rv1536 | -2.4 | 0.002 | isoleucyl-tRNA synthetase iles |
| Rv1591 | -2.4 | 0.028 | probable transmembrane protein |
| Rv1620c | 2.6 | 0.050 | probable 'component linked with the assembly of cytochrome' transport transmembrane atp-binding protein abc transporter cydC |
| Rv1623c | 3.8 | 0.005 | probable integral membrane cytochrome d ubiquinol oxidase (subunit i) cydA (cytochrome bD-i oxidase subunit i) |
| Rv1870c | -2.3 | 0.022 | conserved hypothetical protein |
| Rv1954c | -2.2 | 0.003 | hypothetical protein |
| Rv1955 | -2.0 | 0.013 | possible antitoxin higA1 |
| Rv2010 | -2.5 | 0.003 | possible toxin vapC15 |
| Rv2055c | 4.0 | 0.047 | probable ribosomal protein S18 rpsR2 |
| Rv2057c | 7.0 | 0.017 | probable ribosomal protein L33 |
| Rv2143 | -2.0 | 0.023 | conserved hypothetical protein |
| Rv2243 | -2.1 | 0.002 | malonyl coa-acyl carrier protein transacylase fabd (malonyl coa:acpM acyltransferase) (mct) |
| Rv2251 | -2.3 | 0.036 | possible flavoprotein |
| Rv2252 | -2.2 | 0.050 | possible diacylglycerol kinase |
| Rv2380c | 2.0 | 0.050 | peptide synthetase mbte (peptide synthase) |
| Rv2386c | 2.2 | 0.024 | putative isochorismate synthase mbtI |
| Rv2396 | -2.2 | 0.033 | PE-PGRS family protein |
| Rv2397c | -2.6 | 0.003 | probable sulfate-transport atp-binding protein abc transporter cysA1 |
| Rv2398c | -2.6 | 0.002 | probable sulfate-transport integral membrane protein abc transporter cysW |
| Rv2413c | -2.8 | 0.001 | conserved hypothetical protein |
| Rv2416c | -5.3 | 0.002 | enhanced intracellular survival protein eis, gcn5-related n-acetyltransferase |
| Rv2440c | -2.8 | 0.029 | probable gtp1/obg-family gtp-binding protein obg |
| Rv2450c | -6.3 | 0.002 | probable resuscitation-promoting factor rpfE |
| Rv2516c | -2.5 | 0.030 | hypothetical protein |
| Rv2660c | -4.7 | 0.004 | hypothetical protein |
| Rv2661c | -3.2 | 0.037 | hypothetical protein |
| Rv2699c | -2.4 | 0.006 | conserved hypothetical protein |
| Rv2725c | -2.1 | 0.050 | probable gtp-binding protein hflX |
| Rv2784c | -2.8 | 0.006 | probable lipoprotein lppU |
| Rv2873 | -2.5 | 0.008 | cell surface lipoprotein mpt83 (lipoprotein p23) |
| Rv2876 | -2.4 | 0.024 | possible conserved transmembrane protein |
| Rv2877c | -3.0 | 0.004 | probable conserved integral membrane protein |
| Rv2930 | -2.0 | 0.010 | fatty-acid-coa ligase fadd26 (fatty-acid-coa synthetase) (fatty-acid-coa synthase) |
| Rv2989 | -2.4 | 0.021 | probable transcriptional regulatory protein |
| Rv3003c | -2.2 | 0.005 | probable acetolactate synthase (large subunit) ilvB1 (acetohydroxy-acid synthase) |
| Rv3019c | 2.5 | 0.024 | secreted esat-6 like protein esxR (tb10.3) (esat-6 like protein 9) |
| Rv3020c | 2.4 | 0.050 | esat-6 like protein esxS |
| Rv3095 | -3.4 | 0.019 | hypothetical transcriptional regulatory protein |
| Rv3127 | 2.1 | 0.050 | conserved hypothetical protein |
| Rv3189 | -2.0 | 0.009 | conserved hypothetical protein |
| Rv3196 | -2.2 | 0.050 | conserved hypothetical protein |
| Rv3196A | -2.6 | 0.018 | hypothetical protein |
| Rv3197 | -2.9 | 0.002 | probable conserved atp-binding protein abc transporter |
| Rv3197A | -5.6 | 0.033 | probable transcriptional regulatory protein whiB-like whiB7 |
| Rv3220c | -2.7 | 0.002 | probable two component sensor kinase |
| Rv3230c | -2.4 | 0.050 | hypothetical oxidoreductase |
| Rv3290c | -2.7 | 0.006 | probable l-lysine-epsilon aminotransferase lat (l-lysine aminotransferase) (lysine 6-aminotransferase) |
| Rv3454 | -2.1 | 0.050 | probable conserved integral membrane protein |
| Rv3463 | -2.2 | 0.050 | conserved hypothetical protein |
| Rv3686c | -2.2 | 0.017 | conserved hypothetical protein |
| Rv3788 | -2.0 | 0.050 | hypothetical protein |

Supplementary Table 2.

Gene list 2. Genes showing at least two-fold up- or down-regulation on overexpression of Rv0805, compared to overexpression of Rv0805N97A (p-value < 0.05). Negative numbers represent down-regulation in the Rv0805 overexpressing strain compared to Rv0805N97A overexpressing strain.

| Gene | Fold Change | p-value | Gene function / annotation |
| --- | --- | --- | --- |
| Rv0003 | -2.1 | 0.002 | dna replication and repair protein recF (single-strand dna binding protein) |
| Rv0004 | -2.0 | 0.026 | conserved hypothetical protein |
| Rv0079 | 10.5 | 0.003 | hypothetical protein |
| Rv0166 | -2.1 | 0.030 | probable fatty-acid-coa ligase fadD5 (fatty-acid-coa synthetase) (fatty-acid-coa synthase) |
| Rv0197 | -2.4 | 0.015 | possible oxidoreductase |
| Rv0211 | 2.2 | 0.012 | probable iron-regulated phosphoenolpyruvate carboxykinase [gtp] pckA (phosphoenolpyruvate carboxylase) (pepCK)(pep carboxykinase) |
| Rv0286 | 2.0 | 0.000 | PPE family protein |
| Rv0287 | 2.1 | 0.009 | esat-6 like protein esxG (conserved hypothetical protein tb9.8) |
| Rv0288 | 2.3 | 0.027 | low molecular weight protein antigen 7 esxH (10 kda antigen) (cfp-7) (protein tb10.4) |
| Rv0289 | 2.1 | 0.000 | esx-1 secretion-associated protein espG3 |
| Rv0290 | 2.1 | 0.031 | esx conserved component eccD3. probable transmembrane protein |
| Rv0315 | -2.1 | 0.004 | possible beta-1,3-glucanase precursor |
| Rv0316 | -2.3 | 0.000 | possible muconolactone isomerase |
| Rv0327c | -2.1 | 0.018 | possible cytochrome p450 135a1 cyp135a1 |
| Rv0345 | -2.0 | 0.005 | conserved hypothetical protein |
| Rv0695 | -2.4 | 0.007 | conserved hypothetical protein |
| Rv0701 | -2.1 | 0.002 | probable 50s ribosomal protein l3 rplC |
| Rv0702 | -2.1 | 0.012 | probable 50s ribosomal protein l4 rplD |
| Rv0703 | -2.1 | 0.012 | probable 50s ribosomal protein l23 rplW |
| Rv0713 | -2.0 | 0.021 | probable conserved transmembrane protein |
| Rv0715 | -2.0 | 0.017 | probable 50s ribosomal protein l24 rplX |
| Rv0719 | -2.0 | 0.006 | probable 50s ribosomal protein l6 rplF |
| Rv0782 | -2.2 | 0.019 | probable protease ii ptrBb [second part] (oligopeptidase b) |
| Rv0839 | -2.5 | 0.033 | conserved hypothetical protein |
| Rv0840c | -2.1 | 0.002 | probable proline iminopeptidase pip (prolyl aminopeptidase) (pap) |
| Rv0846c | -2.0 | 0.000 | probable oxidase |
| Rv0850 | -2.1 | 0.003 | putative transposase (fragment) |
| Rv0891c | -2.1 | 0.002 | possible transcriptional regulatory protein |
| Rv0892 | -2.3 | 0.036 | probable monooxygenase |
| Rv0967 | 2.1 | 0.051 | coPPEr-sensitive operon repressor csoR |
| Rv1047 | -2.5 | 0.002 | probable transposase |
| Rv1051c | -2.0 | 0.001 | conserved hypothetical protein |
| Rv1181 | -2.1 | 0.002 | probable polyketide beta-ketoacyl synthase pks4 |
| Rv1199c | -2.5 | 0.028 | possible transposase |
| Rv1482c | -2.0 | 0.004 | conserved hypothetical protein |
| Rv1504c | -2.2 | 0.001 | conserved hypothetical protein |
| Rv1738 | 7.3 | 0.043 | conserved hypothetical protein |
| Rv1757c | -2.2 | 0.006 | putative transposase for insertion sequence element is6110 (fragment) |
| Rv1777 | -2.1 | 0.002 | probable cytochrome p450 144 cyp144 |
| Rv1813c | 5.0 | 0.045 | conserved hypothetical protein |
| Rv1917c | -2.0 | 0.005 | PPE family protein |
| Rv1918c | -2.1 | 0.002 | PPE family protein |
| Rv1979c | -2.4 | 0.016 | possible conserved permease |
| Rv2007c | 5.2 | 0.008 | probable ferredoxin fdxA |
| Rv2030c | 7.6 | 0.009 | conserved hypothetical protein |
| Rv2031c | 10.1 | 0.028 | heat shock protein hspX (alpha-crystallin homolog) (14 kda antigen) (hsp16.3) |
| Rv2032 | 4.2 | 0.008 | conserved hypothetical protein acg |
| Rv2055c | 2.5 | 0.016 | probable ribosomal protein s18 rpsR2 |
| Rv2056c | 3.6 | 0.035 | probable ribosomal protein s14 rpsN2 |
| Rv2057c | 3.6 | 0.007 | probable ribosomal protein L33 |
| Rv2189c | -2.1 | 0.003 | conserved hypothetical protein |
| Rv2276 | -2.4 | 0.003 | cytochrome p450 121 cyp121 |
| Rv2278 | -2.2 | 0.022 | putative transposase for insertion sequence element is6110 (fragment) |
| Rv2331A | -2.2 | 0.005 | hypothetical protein |
| Rv2380c | 2.1 | 0.010 | peptide synthetase mbtE (peptide synthase) |
| Rv2381c | 2.2 | 0.008 | polyketide synthetase mbtD (polyketide synthase) |
| Rv2383c | 2.3 | 0.004 | phenyloxazoline synthase mbtB (phenyloxazoline synthetase) |
| Rv2386c | 2.2 | 0.003 | putative isochorismate synthase mbtI |
| Rv2430c | -2.1 | 0.005 | PPE family protein |
| Rv2499c | -2.0 | 0.011 | possible oxidase regulatory-related protein |
| Rv2501c | -2.4 | 0.013 | probable acetyl-/propionyl-coenzyme a carboxylase alpha chain (alpha subunit) acca1: biotin carboxylase + biotin carboxyl carrier protein (bccP) |
| Rv2503c | -3.5 | 0.018 | probable succinyl-coa:3-ketoacid-coenzyme a transferase (beta subunit) scoB (3-oxo-acid:coa transferase) (oxcT b) (succinyl coa:3-oxoacid coa-transferase) |
| Rv2504c | -3.1 | 0.008 | probable succinyl-coa:3-ketoacid-coenzyme a transferase (alpha subunit) scoA (3-oxo acid:coa transferase) (oxcT a) (succinyl-coa:3-oxoacid-coenzyme a transferase) |
| Rv2512c | -2.5 | 0.002 | transposase for insertion sequence element is1081 |
| Rv2666 | -2.3 | 0.001 | probable transposase for insertion sequence element is1081 (fragment) |
| Rv2815c | -2.2 | 0.002 | probable transposase |
| Rv2874 | -2.1 | 0.011 | possible integral membrane c-type cytochrome biogenesis protein dipZ |
| Rv2892c | -2.0 | 0.005 | PPE family protein |
| Rv2949c | -2.2 | 0.013 | chorismate pyruvate lyase |
| Rv2959c | -2.3 | 0.019 | possible methyltransferase (methylase) |
| Rv2987c | -3.4 | 0.037 | probable 3-isopropylmalate dehydratase (small subunit) leud (isopropylmalate isomerase) (alpha-ipm isomerase) (ipmI) |
| Rv3019c | 2.2 | 0.045 | secreted esat-6 like protein esxR (tb10.3) (esat-6 like protein 9) |
| Rv3020c | 2.3 | 0.048 | esat-6 like protein esxS |
| Rv3023c | -2.3 | 0.003 | probable transposase |
| Rv3094c | -2.0 | 0.000 | conserved hypothetical protein |
| Rv3095 | -2.1 | 0.005 | hypothetical transcriptional regulatory protein |
| Rv3097c | -2.1 | 0.004 | PE-PGRS family protein, triacylglycerol lipase (esterase/lipase) (triglyceride lipase) (tributyrase) |
| Rv3115 | -2.3 | 0.002 | probable transposase |
| Rv3127 | 5.6 | 0.002 | conserved hypothetical protein |
| Rv3131 | 8.5 | 0.029 | conserved hypothetical protein |
| Rv3133c | 3.7 | 0.012 | two component transcriptional regulatory protein devR (probably luxr/uhpa-family) |
| Rv3134c | 3.5 | 0.033 | universal stress protein family protein |
| Rv3139 | 2.1 | 0.047 | probable acyl-coa dehydrogenase fadE24 |
| Rv3140 | 2.3 | 0.050 | probable acyl-coa dehydrogenase fadE23 |
| Rv3160c | -2.0 | 0.004 | possible transcriptional regulatory protein (probably tetR-family) |
| Rv3229c | 3.1 | 0.034 | possible linoleoyl-coa desaturase (delta(6)-desaturase) |
| Rv3360 | -2.2 | 0.003 | conserved hypothetical protein |
| Rv3476c | -2.2 | 0.009 | probable dicarboxylic acid transport integral membrane protein kgtP (dicarboxylate transporter) |
| Rv3485c | -2.1 | 0.024 | probable short-chain type dehydrogenase/reductase |
| Rv3486 | -2.0 | 0.001 | conserved hypothetical protein |
| Rv3646c | -2.1 | 0.026 | dna topoisomerase I topA (omega-protein) (relaxing enzyme) (untwisting enzyme) (swivelase) (type I dna topoisomerase) (nicking-closing enzyme) (topo I) |
| Rv3784 | -2.1 | 0.020 | possible dtdp-glucose 4,6-dehydratase |
| Rv3919c | -2.0 | 0.019 | probable glucose-inhibited division protein b gid |
| Rv3921c | -2.1 | 0.038 | probable conserved transmembrane protein |
| Rv3922c | -2.0 | 0.028 | possible hemolysin |

Supplementary Table 3.

Gene list 3. Genes showing at least two-fold up- or down-regulation on overexpression of Rv0805, compared to overexpression of Rv0805(1-278)N97A (p-value < 0.05). Negative numbers represent down-regulation in the Rv0805 overexpressing strain compared to Rv0805(1-278)N97A overexpressing strain.

| Gene | Fold change | p-value | Gene function / annotation |
| --- | --- | --- | --- |
| Rv0047c | -2.4 | 0.025 | conserved hypothetical protein |
| Rv0058 | 2.1 | 0.015 | probable replicative dna helicase dnaB |
| Rv0184 | 2.1 | 0.052 | conserved hypothetical protein |
| Rv0264c | -2.3 | 0.020 | conserved hypothetical protein |
| Rv0405 | 2.1 | 0.042 | probable membrane bound polyketide synthase pks6 |
| Rv0467 | -2.2 | 0.035 | isocitrate lyase icl (isocitrase) (isocitratase) |
| Rv0485 | -2.4 | 0.023 | possible transcriptional regulatory protein |
| Rv0576 | -4.9 | 0.001 | probable methyltransferase/methylase |
| Rv0577 | -2.1 | 0.033 | conserved hypothetical protein tb27.3 |
| Rv0606 | 2.1 | 0.046 | possible transposase (fragment) |
| Rv0607 | 2.3 | 0.048 | hypothetical protein |
| Rv0654 | -2.9 | 0.011 | probable dioxygenase |
| Rv0829 | 2.0 | 0.054 | possible transposase (fragment) |
| Rv0921 | 2.2 | 0.042 | possible resolvase |
| Rv0978c | -2.2 | 0.068 | PE-PGRS family protein |
| Rv0980c | -2.2 | 0.047 | PE-PGRS family protein |
| Rv0990c | -2.0 | 0.049 | hypothetical protein |
| Rv1014c | -2.1 | 0.041 | probable peptidyl-trna hydrolase pth |
| Rv1015c | -2.6 | 0.006 | probable 50s ribosomal protein L25 rplY |
| Rv1057 | -9.5 | 0.001 | conserved hypothetical protein |
| Rv1058 | -2.7 | 0.008 | probable medium chain fatty-acid-coa ligase fadD14 (fatty-acid-coa synthetase) (fatty-acid-coa synthase) |
| Rv1129c | -5.8 | 0.001 | probable transcriptional regulator protein |
| Rv1130 | -4.5 | 0.001 | possible methylcitrate dehydratase prpD |
| Rv1144 | -3.0 | 0.007 | probable short-chain type dehydrogenase/reductase |
| Rv1169c | -2.9 | 0.003 | PE family protein. possible lipase. |
| Rv1187 | -2.1 | 0.041 | probable pyrroline-5-carboxylate dehydrogenase rocA |
| Rv1221 | -2.2 | 0.011 | alternative rna polymerase sigma factor sigE |
| Rv1258c | -3.0 | 0.019 | probable conserved integral membrane transport protein |
| Rv1265 | -2.0 | 0.049 | hypothetical protein |
| Rv1266c | -2.2 | 0.041 | probable transmembrane serine/threonine-protein kinase h pknH (protein kinase h) (stpK h) |
| Rv1267c | -2.1 | 0.047 | probable transcriptional regulatory protein embR |
| Rv1277 | 2.1 | 0.020 | conserved hypothetical protein |
| Rv1279 | 2.2 | 0.008 | probable dehydrogenase fad flavoprotein gmc oxidoreductase |
| Rv1285 | -2.3 | 0.033 | probable sulfate adenylyltransferase subunit 2 cysD |
| Rv1318c | -4.2 | 0.002 | possible adenylate cyclase (atp pyrophosphate-lyase) (adenylyl cyclase) |
| Rv1319c | -2.6 | 0.010 | possible adenylate cyclase (atp pyrophosphate-lyase) (adenylyl cyclase) |
| Rv1405c | -2.5 | 0.015 | putative methyltransferase |
| Rv1460 | -6.4 | 0.001 | probable transcriptional regulatory protein |
| Rv1483 | -3.0 | 0.009 | 3-oxoacyl-[acyl-carrier protein] reductase fabG1 (3-ketoacyl-acyl carrier protein reductase) (mycolic acid biosynthesis a protein) |
| Rv1484 | -2.5 | 0.027 | NADH-dependent enoyl-[acyl-carrier-protein] reductase inhA (NADH-dependent enoyl-ACP reductase) |
| Rv1535 | -2.0 | 0.044 | hypothetical protein |
| Rv1536 | -2.3 | 0.018 | isoleucyl-trna synthetase ileS |
| Rv1593c | -2.1 | 0.033 | conserved hypothetical protein |
| Rv1805c | -3.0 | 0.014 | hypothetical protein |
| Rv1954c | -2.2 | 0.041 | hypothetical protein |
| Rv1988 | -2.6 | 0.032 | probable methyltransferase |
| Rv2014 | 2.2 | 0.053 | possible transposase |
| Rv2035 | -2.2 | 0.026 | conserved hypothetical protein |
| Rv2191 | 2.5 | 0.022 | conserved hypothetical protein |
| Rv2396 | -2.4 | 0.019 | PE-PGRS family protein |
| Rv2397c | -2.1 | 0.055 | probable sulfate-transport atp-binding protein abc transporter cysA1 |
| Rv2414c | -3.6 | 0.017 | conserved hypothetical protein |
| Rv2415c | -3.2 | 0.007 | conserved hypothetical protein |
| Rv2416c | -4.8 | 0.002 | enhanced intracellular survival protein eis, gcn5-related n-acetyltransferase |
| Rv2439c | -2.0 | 0.047 | probable glutamate 5-kinase protein proB (gamma-glutamyl kinase) (gk) |
| Rv2440c | -4.2 | 0.002 | probable gtp1/obg-family gtp-binding protein obg |
| Rv2442c | -2.0 | 0.053 | probable 50s ribosomal protein L21 rplU |
| Rv2450c | -18.9 | 0.000 | probable resuscitation-promoting factor rpfE |
| Rv2466c | -2.3 | 0.026 | conserved hypothetical protein |
| Rv2515c | -2.4 | 0.050 | conserved hypothetical protein |
| Rv2516c | -2.9 | 0.011 | hypothetical protein |
| Rv2517c | -2.0 | 0.022 | hypothetical protein |
| Rv2594c | 2.0 | 0.046 | probable crossover junction endodeoxyribonuclease ruvC (holliday junction nuclease) (holliday junction resolvase) |
| Rv2660c | -6.1 | 0.001 | hypothetical protein |
| Rv2661c | -4.0 | 0.004 | hypothetical protein |
| Rv2667 | -2.5 | 0.014 | possible atp-dependent protease atp-binding subunit clpC2 |
| Rv2706c | -2.3 | 0.023 | hypothetical protein |
| Rv2707 | -2.1 | 0.041 | probable conserved transmembrane alanine and leucine rich protein |
| Rv2791c | 2.0 | 0.046 | probable transposase |
| Rv2792c | 2.0 | 0.049 | possible resolvase |
| Rv2885c | 2.1 | 0.032 | probable transposase |
| Rv2913c | -2.0 | 0.034 | possible d-amino acid aminohydrolase (d-amino acid hydrolase) |
| Rv2978c | 2.0 | 0.048 | probable transposase |
| Rv3191c | 2.2 | 0.055 | probable transposase |
| Rv3196 | -3.5 | 0.008 | conserved hypothetical protein |
| Rv3196A | -2.2 | 0.044 | hypothetical protein |
| Rv3197 | -5.0 | 0.001 | probable conserved ATP-binding protein ABC transporter |
| Rv3197A | -11.3 | 0.000 | probable transcriptional regulatory protein whiB-like whiB7 |
| Rv3201c | 2.6 | 0.011 | probable ATP-dependent DNA helicase |
| Rv3202c | 2.4 | 0.012 | possible ATP-dependent DNA helicase |
| Rv3219 | -2.2 | 0.038 | probable transcriptional regulatory protein whiB-like whiB1 |
| Rv3290c | -3.5 | 0.012 | probable l-lysine-epsilon aminotransferase lat (l-lysine aminotransferase) (lysine 6-aminotransferase) |
| Rv3517 | 2.3 | 0.033 | conserved hypothetical protein |
| Rv3555c | 2.0 | 0.047 | conserved hypothetical protein |
| Rv3585 | 2.3 | 0.027 | DNA repair protein radA (dna repair protein sms) |
| Rv3666c | -2.4 | 0.017 | probable periplasmic dipeptide-binding lipoprotein dppA |
| Rv3827c | 2.0 | 0.073 | possible transposase |

Supplementary table 4.

Gene set A. Genes dysregulated in Gene list 1 and not in Gene list 2.

| Gene | Fold | Function |
| --- | --- | --- |
| Rv0047c | -2.7 | conserved hypothetical protein |
| Rv0053 | -2.9 | probable 30s ribosomal protein s6 rpsF |
| Rv0251c | -2.1 | heat shock protein hsp (heat-stress-induced ribosome-binding protein a) |
| Rv0302 | -3.0 | probable transcriptional regulatory protein (probably tetR/acrR-family) |
| Rv0337c | -2.2 | probable aspartate aminotransferase aspC (transaminase a) (aspAT) |
| Rv0341 | -2.8 | isoniazid inducible gene protein iniB |
| Rv0342 | -2.6 | isoniazid inducible gene protein iniA |
| Rv0465c | -2.3 | probable transcriptional regulatory protein |
| Rv0467 | -2.5 | isocitrate lyase icl (isocitrase) (isocitratase) |
| Rv0477 | -2.0 | possible conserved secreted protein |
| Rv0484c | -2.3 | probable short-chain type oxidoreductase |
| Rv0485 | -2.2 | possible transcriptional regulatory protein |
| Rv0576 | -3.1 | probable transcriptional regulatory protein (possibly arsR-family) |
| Rv0651 | -2.6 | probable 50s ribosomal protein l10 rplJ |
| Rv0654 | -3.2 | probable dioxygenase |
| Rv0656c | -2.1 | possible toxin vapC6 |
| Rv0714 | -3.3 | probable 50s ribosomal protein L14 rplN |
| Rv0749 | -2.2 | conserved hypothetical protein with pin domain |
| Rv0809 | -2.1 | probable phosphoribosylformylglycinamidine cyclo-ligase purm (airS) (phosphoribosyl-aminoimidazole synthetase) (air synthase) |
| Rv0812 | -2.2 | probable amino acid aminotransferase |
| Rv0969 | 2.4 | probable metal cation transporter p-type atpase ctpV |
| Rv0980c | -3.7 | pe-pgrs family protein |
| Rv1014c | -2.6 | probable peptidyl-tRNA hydrolase pth |
| Rv1015c | -4.7 | probable 50s ribosomal protein l25 rplY |
| Rv1046c | -2.1 | hypothetical protein |
| Rv1057 | -2.6 | conserved hypothetical protein |
| Rv1079 | -2.7 | probable cystathionine gamma-synthase metb (cgs) (o-succinylhomoserine [thiol]-lyase) |
| Rv1080c | -2.5 | probable transcription elongation factor greA (transcript cleavage factor greA) |
| Rv1103c | -2.1 | possible antitoxin mazE3 |
| Rv1129c | -5.7 | probable transcriptional regulator protein |
| Rv1130 | -9.5 | possible methylcitrate dehydratase prpD |
| Rv1131 | -5.3 | probable methylcitrate synthase prpC |
| Rv1144 | -2.6 | probable short-chain type dehydrogenase/reductase |
| Rv1169c | -3.6 | PE family protein. possible lipase. |
| Rv1305 | 2.0 | probable ATP synthase C chain atpE (lipid-binding protein) (dicyclohexylcarbodiimide-binding protein) |
| Rv1317c | -2.8 | probable ada regulatory protein alkA (regulatory protein of adaptative response) (methylated-DNA--protein-cysteine methyltransferase) (o-6-methylguanine-DNA alkyltransferase) (o-6-methylguanine-DNA methyltransferase) (3-methyladenine DNA glycosylase ii) |
| Rv1318c | -3.4 | possible adenylate cyclase (ATP pyrophosphate-lyase) (adenylyl cyclase) |
| Rv1319c | -3.1 | possible adenylate cyclase (ATP pyrophosphate-lyase) (adenylyl cyclase) |
| Rv1374c | -2.0 | hypothetical protein |
| Rv1405c | -2.0 | putative methyltransferase |
| Rv1460 | -3.6 | probable transcriptional regulatory protein |
| Rv1485 | -2.1 | ferrochelatase hemZ (protoheme ferro-lyase) (heme synthetase) |
| Rv1533 | -2.3 | conserved hypothetical protein |
| Rv1535 | -2.1 | hypothetical protein |
| Rv1536 | -2.4 | isoleucyl-tRNA synthetase ileS |
| Rv1591 | -2.4 | probable transmembrane protein |
| Rv1620c | 2.6 | probable 'component linked with the assembly of cytochrome' transport transmembrane ATP-binding protein ABC transporter cydC |
| Rv1623c | 3.8 | probable integral membrane cytochrome d ubiquinol oxidase (subunit i) cydA (cytochrome BD-I oxidase subunit I) |
| Rv1870c | -2.3 | conserved hypothetical protein |
| Rv1954c | -2.2 | hypothetical protein |
| Rv1955 | -2.0 | possible antitoxin higA1 |
| Rv2010 | -2.5 | possible toxin vapC15 |
| Rv2143 | -2.0 | conserved hypothetical protein |
| Rv2243 | -2.1 | malonyl coa-acyl carrier protein transacylase fabD (malonyl coa:acpM acyltransferase) (mct) |
| Rv2251 | -2.3 | possible flavoprotein |
| Rv2252 | -2.2 | possible diacylglycerol kinase |
| Rv2396 | -2.2 | PE-PGRS family protein |
| Rv2397c | -2.6 | probable sulfate-transport ATP-binding protein ABC transporter cysA1 |
| Rv2398c | -2.6 | probable sulfate-transport integral membrane protein ABC transporter cysW |
| Rv2413c | -2.8 | conserved hypothetical protein |
| Rv2416c | -5.3 | enhanced intracellular survival protein eis, gcn5-related N-acetyltransferase |
| Rv2440c | -2.8 | probable gtp1/obg-family gtp-binding protein obg |
| Rv2450c | -6.3 | probable resuscitation-promoting factor rpfE |
| Rv2516c | -2.5 | hypothetical protein |
| Rv2660c | -4.7 | hypothetical protein |
| Rv2661c | -3.2 | hypothetical protein |
| Rv2699c | -2.4 | conserved hypothetical protein |
| Rv2725c | -2.1 | probable GTP-binding protein hflX |
| Rv2784c | -2.8 | probable lipoprotein lppU |
| Rv2873 | -2.5 | cell surface lipoprotein mpt83 (lipoprotein p23) |
| Rv2876 | -2.4 | possible conserved transmembrane protein |
| Rv2877c | -3.0 | probable conserved integral membrane protein |
| Rv2930 | -2.0 | fatty-acid-coa ligase fadD26 (fatty-acid-coa synthetase) (fatty-acid-coa synthase) |
| Rv2989 | -2.4 | probable transcriptional regulatory protein |
| Rv3003c | -2.2 | probable acetolactate synthase (large subunit) ilvB1 (acetohydroxy-acid synthase) |
| Rv3189 | -2.0 | conserved hypothetical protein |
| Rv3196 | -2.2 | conserved hypothetical protein |
| Rv3196A | -2.6 | hypothetical protein |
| Rv3197 | -2.9 | probable conserved ATP-binding protein ABC transporter |
| Rv3197A | -5.6 | probable transcriptional regulatory protein whiB-like whiB7 |
| Rv3220c | -2.7 | probable two component sensor kinase |
| Rv3230c | -2.4 | hypothetical oxidoreductase |
| Rv3290c | -2.7 | probable l-lysine-epsilon aminotransferase lat (l-lysine aminotransferase) (lysine 6-aminotransferase) |
| Rv3454 | -2.1 | probable conserved integral membrane protein |
| Rv3463 | -2.2 | conserved hypothetical protein |
| Rv3686c | -2.2 | conserved hypothetical protein |
| Rv3788 | -2.0 | hypothetical protein |

Supplementary Table 5.

Gene set B. Genes dysregulated in Gene list 1 and Gene list 2

| Gene | Fold (Gene List 1) | Fold (Gene List 2) | Function |
| --- | --- | --- | --- |
| Rv0079 | 2.6 | 10.5 | hypothetical protein |
| Rv0197 | 2.0 | -2.4 | possible oxidoreductase |
| Rv0286 | 2.4 | 2.0 | PPE family protein |
| Rv0287 | 2.5 | 2.1 | esat-6 like protein esxG (conserved hypothetical protein tb9.8) |
| Rv0288 | 2.7 | 2.3 | low molecular weight protein antigen 7 esxH (10 kda antigen) (cfp-7) (protein tb10.4) |
| Rv0289 | 2.7 | 2.1 | esx-1 secretion-associated protein espG3 |
| Rv0290 | 2.5 | 2.1 | esx conserved component eccD3. probable transmembrane protein |
| Rv0316 | -2.2 | -2.3 | possible muconolactone isomerase |
| Rv0701 | -2.3 | -2.1 | probable 50s ribosomal protein L3 rplC |
| Rv0715 | -2.3 | -2.0 | probable 50s ribosomal protein L24 rplX |
| Rv0839 | -3.0 | -2.5 | conserved hypothetical protein |
| Rv0967 | 2.4 | 2.1 | copper-sensitive operon repressor csoR |
| Rv2055c | 4.0 | 2.5 | probable ribosomal protein S18 rpsR2 |
| Rv2057c | 7.0 | 3.6 | probable ribosomal protein L33 |
| Rv2380c | 2.0 | 2.1 | peptide synthetase mbtE (peptide synthase) |
| Rv2386c | 2.2 | 2.2 | putative isochorismate synthase mbtI |
| Rv3019c | 2.5 | 2.2 | secreted esat-6 like protein esxR (tb10.3) (esat-6 like protein 9) |
| Rv3020c | 2.4 | 2.3 | esat-6 like protein esxS |
| Rv3095 | -3.4 | -2.1 | hypothetical transcriptional regulatory protein |
| Rv3127 | 2.1 | 5.6 | conserved hypothetical protein |

Supplementary table 6.

Genes dysregulated in Gene list 2 and not in gene list 1.

| Gene | Fold | Function |
| --- | --- | --- |
| Rv2031c | 10.1 | heat shock protein hspX (alpha-crystallin homolog) (14 kda antigen) (hsp16.3) |
| Rv3131 | 8.5 | conserved hypothetical protein |
| Rv2030c | 7.6 | conserved hypothetical protein |
| Rv1738 | 7.3 | conserved hypothetical protein |
| Rv2007c | 5.2 | probable ferredoxin fdxA |
| Rv1813c | 5.0 | conserved hypothetical protein |
| Rv2032 | 4.2 | conserved hypothetical protein acg |
| Rv3133c | 3.7 | two component transcriptional regulatory protein devR (probably luxr/uhpa-family) |
| Rv2056c | 3.6 | probable ribosomal protein S14 rpsN2 |
| Rv3134c | 3.5 | universal stress protein family protein |
| Rv3229c | 3.1 | possible linoleoyl-coa desaturase (delta(6)-desaturase) |
| Rv2383c | 2.3 | phenyloxazoline synthase mbtB (phenyloxazoline synthetase) |
| Rv3140 | 2.3 | probable acyl-coa dehydrogenase fadE23 |
| Rv2381c | 2.2 | polyketide synthetase mbtD (polyketide synthase) |
| Rv0211 | 2.2 | probable iron-regulated phosphoenolpyruvate carboxykinase [GTP] pckA (phosphoenolpyruvate carboxylase) (pepCK)(pep carboxykinase) |
| Rv3139 | 2.1 | probable acyl-coa dehydrogenase fadE24 |
| Rv1979c | -2.4 | possible conserved permease |
| Rv3476c | -2.2 | probable dicarboxylic acid transport integral membrane protein kgtP (dicarboxylate transporter) |
| Rv3921c | -2.1 | probable conserved transmembrane protein |
| Rv0713 | -2.0 | probable conserved transmembrane protein |
| Rv3919c | -2.0 | probable glucose-inhibited division protein b gid |
| Rv0695 | -2.4 | conserved hypothetical protein |
| Rv1504c | -2.2 | conserved hypothetical protein |
| Rv3360 | -2.2 | conserved hypothetical protein |
| Rv2331A | -2.2 | hypothetical protein |
| Rv2189c | -2.1 | conserved hypothetical protein |
| Rv0004 | -2.0 | conserved hypothetical protein |
| Rv3094c | -2.0 | conserved hypothetical protein |
| Rv3486 | -2.0 | conserved hypothetical protein |
| Rv1482c | -2.0 | conserved hypothetical protein |
| Rv1051c | -2.0 | conserved hypothetical protein |
| Rv0345 | -2.0 | conserved hypothetical protein |
| Rv0003 | -2.1 | DNA replication and repair protein recF (single-strand DNA binding protein) |
| Rv0702 | -2.1 | probable 50s ribosomal protein L4 rplD |
| Rv0703 | -2.1 | probable 50s ribosomal protein L23 rplW |
| Rv3646c | -2.1 | DNA topoisomerase I topA (omega-protein) (relaxing enzyme) (untwisting enzyme) (swivelase) (type I DNA topoisomerase) (nicking-closing enzyme) (topo I) |
| Rv0719 | -2.0 | probable 50s ribosomal protein L6 rplF |
| Rv1199c | -2.5 | possible transposase |
| Rv1047 | -2.5 | probable transposase |
| Rv2512c | -2.5 | transposase for insertion sequence element IS1081 |
| Rv3115 | -2.3 | probable transposase |
| Rv2666 | -2.3 | probable transposase for insertion sequence element IS1081 (fragment) |
| Rv2815c | -2.2 | probable transposase |
| Rv1757c | -2.2 | putative transposase for insertion sequence element IS6110 (fragment) |
| Rv2278 | -2.2 | putative transposase for insertion sequence element IS6110 (fragment) |
| Rv0850 | -2.1 | putative transposase (fragment) |
| Rv2987c | -3.4 | probable 3-isopropylmalate dehydratase (small subunit) leuD (isopropylmalate isomerase) (alpha-ipm isomerase) (ipmI) |
| Rv2276 | -2.4 | cytochrome p450 121 cyp121 |
| Rv2959c | -2.3 | possible methyltransferase (methylase) |
| Rv0892 | -2.3 | probable monooxygenase |
| Rv0782 | -2.2 | probable protease II ptrBb [second part] (oligopeptidase b) |
| Rv2949c | -2.2 | chorismate pyruvate lyase |
| Rv0315 | -2.1 | possible beta-1,3-glucanase precursor |
| Rv0840c | -2.1 | probable proline iminopeptidase pip (prolyl aminopeptidase) (pap) |
| Rv0327c | -2.1 | possible cytochrome p450 135a1 cyp135a1 |
| Rv1777 | -2.1 | probable cytochrome p450 144 cyp144 |
| Rv3485c | -2.1 | probable short-chain type dehydrogenase/reductase |
| Rv2874 | -2.1 | possible integral membrane c-type cytochrome biogenesis protein dipZ |
| Rv3784 | -2.1 | possible dtdP-glucose 4,6-dehydratase |
| Rv2499c | -2.0 | possible oxidase regulatory-related protein |
| Rv0846c | -2.0 | probable oxidase |
| Rv2503c | -3.5 | probable succinyl-coa:3-ketoacid-coenzyme a transferase (beta subunit) scoB (3-oxo-acid:coa transferase) (oxcT B) (succinyl coa:3-oxoacid coa-transferase) |
| Rv2504c | -3.1 | probable succinyl-coa:3-ketoacid-coenzyme a transferase (alpha subunit) scoA (3-oxo acid:coa transferase) (oxcT A) (succinyl-coa:3-oxoacid-coenzyme A transferase) |
| Rv2501c | -2.4 | probable acetyl-/propionyl-coenzyme a carboxylase alpha chain (alpha subunit) accA1: biotin carboxylase + biotin carboxyl carrier protein (bccP) |
| Rv1181 | -2.1 | probable polyketide beta-ketoacyl synthase pks4 |
| Rv0166 | -2.1 | probable fatty-acid-coa ligase fadD5 (fatty-acid-coa synthetase) (fatty-acid-coa synthase) |
| Rv1918c | -2.1 | PPE family protein |
| Rv2430c | -2.1 | PPE family protein |
| Rv3097c | -2.1 | PE-PGRS family protein, triacylglycerol lipase (esterase/lipase) (triglyceride lipase) (tributyrase) |
| Rv2892c | -2.0 | PPE family protein |
| Rv1917c | -2.0 | PPE family protein |
| Rv0891c | -2.1 | possible transcriptional regulatory protein |
| Rv3160c | -2.0 | possible transcriptional regulatory protein (probably tetR-family) |
| Rv3922c | -2.0 | possible hemolysin |
